# Acupuncture for Diarrhoea-Predominant Irritable Bowel Syndrome: A Network Meta-Analysis

**DOI:** 10.1155/2018/2890465

**Published:** 2018-05-27

**Authors:** Lingping Zhu, Yunhui Ma, Shasha Ye, Zhiqun Shu

**Affiliations:** ^1^General Practice Department of Shenzhen Longhua District Central Hospital, China; ^2^Zhongshan Hospital of Fudan University, China; ^3^Pudong Institute for Health Development, China

## Abstract

**Background:**

The objective of this study was to compare the efficacy and side effects of acupuncture, sham acupuncture, and drugs in the treatment of diarrhoea-predominant irritable bowel syndrome.

**Methods:**

Randomized controlled trials (RCTs) assessing the effects of acupuncture and drugs were comprehensively retrieved from electronic databases (such as PubMed, Cochrane Library, Embase, CNKI, Wanfang Database, VIP Database, and CBM) up to December 2017. Additional references were obtained from review articles. With document quality evaluations and data extraction, Network Meta-Analysis was performed using a random-effects model under a frequentist framework.

**Results:**

A total of 29 studies (n = 9369) were included; 19 were high-quality studies, and 10 were low-quality studies. NMA showed the following: (1) the ranking of treatments in terms of efficacy in diarrhoea-predominant irritable bowel syndrome is acupuncture, sham acupuncture, pinaverium bromide, alosetron = eluxadoline, ramosetron, and rifaximin; (2) the ranking of treatments in terms of severity of side effects in diarrhoea-predominant irritable bowel syndrome is rifaximin, alosetron, ramosetron = pinaverium bromide, sham acupuncture, and acupuncture; and (3) the treatment of diarrhoea-predominant irritable bowel syndrome includes common acupoints such as ST25, ST36, ST37, SP6, GV20, and EX-HN3.

**Conclusion:**

Acupuncture may improve diarrhoea-predominant irritable bowel syndrome better than drugs and has the fewest side effects. Sham acupuncture may have curative effect except for placebo effect. In the future, it is necessary to perform highly qualified research to prove this result. Pinaverium bromide also has good curative effects with fewer side effects than other drugs.

## 1. Introduction

Irritable bowel syndrome (IBS) is a disease with a high incidence rate, and diarrhoea-predominant irritable bowel syndrome (IBS-D) is a subtype of irritable bowel syndrome with a major clinical manifestation. IBS has a prevalence ranging from 1.1 to 29.2% in the whole population according to the Rome III criteria, with the diarrhoea-predominant type accounting for about 23.4% [1, 2]. Diarrhoea-predominant irritable bowel syndrome (IBS-D) leads to a great deal of trouble [3]. However, the pathogenesis of diarrhoea-predominant irritable bowel syndrome is not yet clear, and its aetiology is complex and may be caused by a variety of factors including visceral allergies, inflammatory responses, heredity, gastrointestinal motility disorders, intestinal infections, and psychosocial factors. In addition, there is a lack of morphological or biochemical abnormalities and other available organic diseases to explain the clinical symptoms [4, 5]. The current treatment methods for IBS-D include drugs and acupuncture treatment; common drugs include pinaverium bromide, eluxadoline, alosetron, ramosetron, rifaximin, and intestinal probiotics. Currently, increasing studies have shown that acupuncture may have some effect on IBS-D, but there are no efficacy comparisons between acupuncture and commonly used oral drugs, and each patient uses different acupuncture points, so we were interested in conducting a systematic review to resolve these two problems.

Now, more and more studies use sham acupuncture as the control of acupuncture. However, there is a debate on whether sham acupuncture has curative effect and to what extent sham acupuncture does affect the final result; this question could be solved with the Network Meta-Analysis.

In this study, by collecting previously published treatments of IBS-D in randomized controlled treatment studies using acupuncture and oral common drugs, we expected to determine the following issues: (1) a ranking of acupuncture and drugs in the treatment of diarrhoea-predominant irritable bowel syndrome; (2) a ranking of acupuncture and drugs in their side effects on diarrhoea-predominant irritable bowel syndrome; (3) the extent to which sham acupuncture does effect the final result; (4) the acupoint distributions used to treat diarrhoea-predominant irritable bowel syndrome.

## 2. Materials and Method

We conducted a standardized report based on the preferred reporting items of the PRISMA statement [6, 7].

### 2.1. Research Methods

We searched PubMed, the Cochrane Library, Embase, and 4 Chinese databases [China National Knowledge Infrastructure (CNKI), Wanfang Database, VIP Database, and Chinese Biomedical Database (CBM)] to conduct a comprehensive database retrieval using a (acupuncture or electro-acupuncture Or acupuncture, Sham Acupuncture, pinaverium bromide, alosetron, eluxadoline, ramosetron, rifaximin), (randomized controlled trials or randomized controlled trials or clinical trials), and (IBS-D) strategy (the retrieval time was from the building of database to 17^th^ October, 2017). In addition, the same search was conducted for the reference reviews and meta-analyses cited in manual searches, with no language restrictions set (searching strategy in Supplementary 1).

### 2.2. Inclusion and Exclusion Criteria

We included randomized controlled trials that met the following eligibility criteria: (i) adult patients; (ii) single drug use; (iii) clinical trials with treatment duration greater than two weeks; (iv) articles that were not comments or commentary; and (v) patients that did not suffer from pregnancy or lactation, peptic ulcer, rectal disease, or liver or other systemic disease and had no previous history of gastroduodenal surgery or brain disease or surgery.

### 2.3. Research Options

Articles were independently screened by two researchers. Initially, NoteExpress software (Beijing Aegean Sea Music Technology Co., Ltd.) was used to delete duplicate records. The remaining summaries and full texts were reviewed on the basis of inclusion and exclusion criteria, and disagreements were resolved through discussion.

### 2.4. Data Extraction and Quality Assessment

Two reviewers (Lingping Zhu and Shasha Ye) independently extracted the relevant information from each eligible study based on a pre-prepared data abstraction sheet. Data included the location and study design of the trials, clinical characteristics, number of patients, patient age, diagnostic methods, treatment duration, outcome data, and side effects. The quality of the included studies was assessed using the Jadad scale, including three items such as randomized (2 points), double-blinded (2 points), and withdrawals and drop-outs (1 point) [8]. A Jadad score of 3 or higher was considered to be high quality. Disagreements were resolved by discussion.

The primary outcome was the number of people who showed effective treatment, with secondary outcomes including side effects and common acupuncture points. Common side effects included constipation and rash.

### 2.5. Data Synthesis and Analysis

The assessments of acupuncture and drug efficacy were based on a combination of the data extracted from the included trials, and then direct and indirect comparisons were used to assess the overall effect of acupuncture and medications. In this meta-analysis of the network, we used a random-effects model in a Bayesian framework. The odds ratio (OR) and 95 % confidence interval (CI) were used to analyse the effects of acupuncture and drugs on the efficacy of diarrhoea-predominant irritable bowel syndrome. CIs with OR> 1.0 indicated high risk, and CIs not containing 1.0 were considered statistically significant. All analyses used the GeMTC package generated by R software [9, 10].

Node-splitting models were used to assess the consistency of the meta-analysis of the network to test whether the results of the direct and indirect comparisons were consistent within the treatment cycle [11]. In the absence of direct or indirect comparison results, the node-split model cannot be executed. Therefore, we use heterogeneity analysis to quantify the degree of heterogeneity of I^2^ calculations. I^2^ > 50 % of the value was considered heterogeneous throughout the experiment. To verify the robustness of the results, sensitivity analyses were performed by examining heterogeneity in each study and then recalculating the overall effect to see if any of the factors could affect the overall effect.

A mesh diagram, contribution graphs, and publication bias tests were drawn using STATA 14.0 software (Stata Corporation, College Station, TX, USA).

## 3. Results

### 3.1. Included Research Features

**Figure 1:**
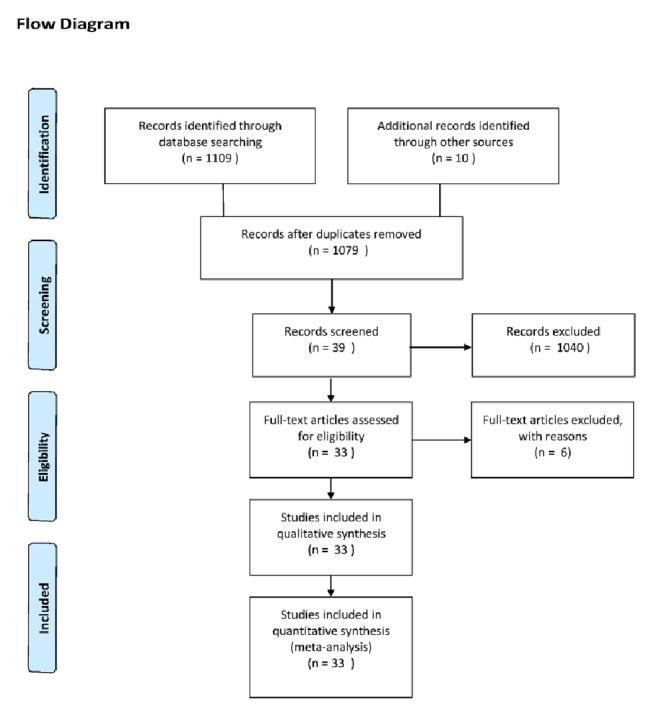
Identification process for eligible trials.

A total of 1119 articles were obtained from the system search. After reviewing the literature, 40 duplicates were deleted. In addition, due to discrepancies in inclusion criteria, 1046 articles were excluded. Finally, a total of 33 trials were identified (Figure 1) and are listed in Table 1 [11–40].

In total, 9712 patients diagnosed with IBS-D/IBS were enrolled in the assessed studies, mean age was between 38 and 46 years, the diagnosis criteria included clinical criteria, ROME I-III, and the treatment duration was from 2 weeks to 48 weeks, mainly between 4 and 12 weeks. The following seven therapeutic methods were included: A: acupuncture; B: eluxadoline; C: pinaverium bromide; D: alosetron; E: ramosetron; F: rifaximin; and G: sham acupuncture; H: placebo (vitamin C, etc.). Documents included 10 articles from China, 9 articles from the United States, 2 papers from France, 2 papers from Canada, 1 paper from United Kingdom, 5 articles from Japan, 1 article from Korea, and 3 articles from multicentre locations. Using the Jadad scale assessment, the overall Jadad score for study quality ranged from 1 to 7, and the median Jadad score was 4 (see Table 1 for details).

### 3.2. Routine Paired Meta-Analysis

**Figure 2:**
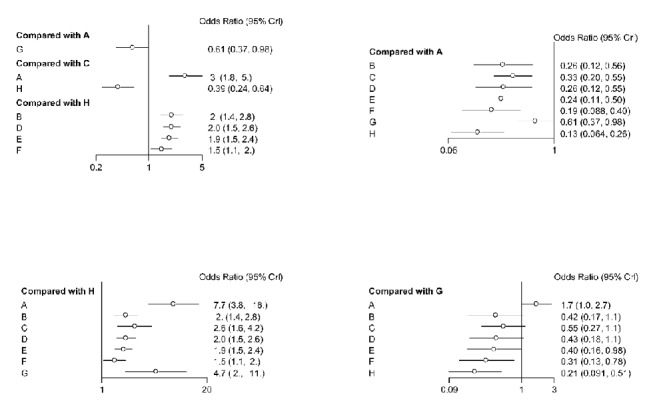
The Forest plot of IBS-D treatment of acupuncture compared with other drugs. A: acupuncture; B: eluxadoline; C: pinaverium bromide; D: alosetron E: ramosetron; F: rifaximin; G: sham acupuncture; H: placebo.

Compared with placebo, acupuncture significantly improved the symptoms of diarrhoea-predominant irritable bowel syndrome (OR: 7.7, 95% CI: 3.8-16.0, I^2^ = 0%) (Figure 2); compared with placebo, sham acupuncture significantly improved the symptoms of diarrhoea-predominant irritable bowel syndrome (OR:4.7, 95% CI: 2.0 to 11.0); compared with placebo, pinaverium bromide significantly improved the symptoms of diarrhoea-predominant irritable bowel syndrome (OR: 2.6, 95% CI: 1.5 to 4.1, I^2^ = 0%) (Figure 2); eluxadoline significantly improved the symptoms of diarrhoea-predominant irritable bowel syndrome compared with placebo (OR: 2.0, 95% CI: 1.4-2.8, I^2^ = 5.3%) (Figure 2); compared with placebo, alosetron also improved the symptoms of diarrhoea-predominant irritable bowel syndrome (OR: 2.0, 95% CI: 1.5-2.6, I^2^ = 53.3%); compared with placebo, ramosetron also improved the symptoms of diarrhoea-predominant irritable bowel syndrome (OR: 1.9, 95% CI: 1.5-2.4, I^2^ = 68.1%); and compared with placebo, rifaximin treatment improved the symptoms of diarrhoea-predominant irritable bowel syndrome (OR: 1.5, 95% CI: 1.0-2.0, I^2^ = 0%) (Figure 2). The efficacy of drugs compared with acupuncture and sham acupuncture was poor (Figure 2).

### 3.3. The Cumulative Probability Ranking

**Figure 3:**
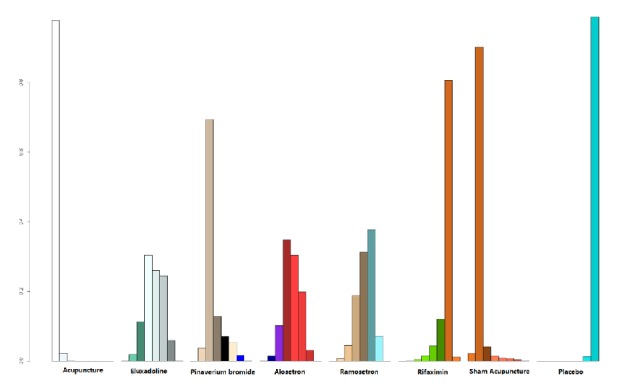
The cumulative probability ranking plot of treatment effect of acupuncture and other drugs on IBS-D.

The cumulative probability ranking of the results for diarrhoea-predominant irritable bowel syndrome patients is as follows: acupuncture, sham acupuncture, pinaverium, alosetron = eluxadoline, ramosetron, and rifaximin. The probability distribution rankings of eluxadoline were equal, so we chose the probability of the closest top rank as its ranking result. The efficacy of acupuncture was much higher than that of other drugs (P = 0.977), while sham acupuncture had a higher drug efficacy (P = 0.90) than pinaverium bromide (P=0.69), alosetron (P = 0.35), eluxadoline (P= 0.30), ramosetron (P=0.31), and rifaximin (P=0.81) (Figure 3, Table 2).

**Figure 4:**
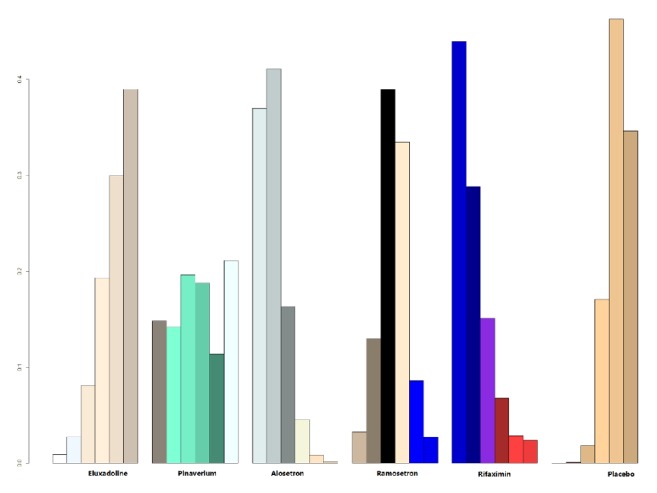
The cumulative probability ranking plot of side effect of drugs on IBS-D.

There were 22 studies that reported side effect data (Table 1); there were no reported side effects from acupuncture, so acupuncture was not included in the analysis. The rest of the reported side effect data contained all other 6 treatment regimens (Table 3). Because the side effects of acupuncture were 0, its side effects were the lowest, followed by other drugs; the smallest side effects were for eluxadoline (P = 0.39) and pinaverium bromide (P = 0.21), and there were more side effects from rifaximin (P = 0.44) than from other drugs. Ramosetron also showed more side effects than alosetron (Figure 4).

### 3.4. Network Plot

**Figure 5:**
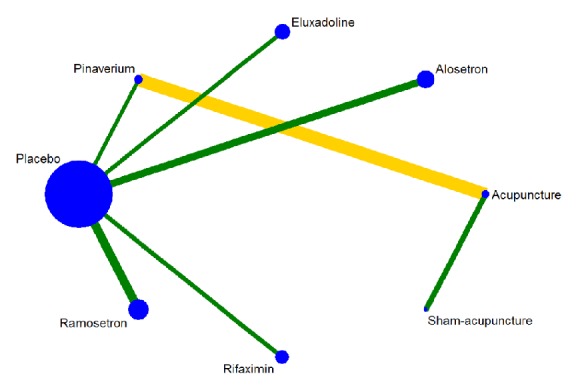
The network plot of all treatment methods: yellow means the low-quality studies, green means the high-quality studies.

We compared all of the included studies and drew network diagrams, with the studies incorporated into quality-based displays on a network map (Figure 5).

### 3.5. Acupuncture Preference Points

In view of the different acupuncture points selected for each study, we selected the most commonly used acupoints, including ST-25, ST-37, ST-36, SP-6, GV-20, and EX-HN3; the use of these 6 acupoints was 4 times more common than other acupoints (Table 4).

### 3.6. Brooks-Gelman-Rubin Diagnostic Plot, Density Plot, Node-Splitting Plot, and Cumulative Contribution Plot

**Figure 6:**
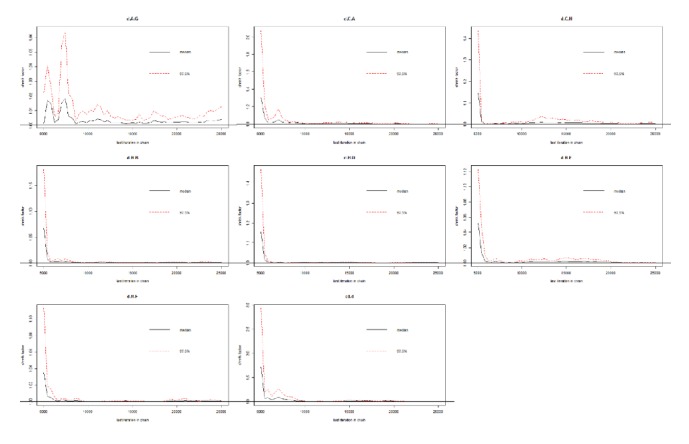
Brooks-Gelman-Rubin diagnostic plot of included studies.

**Figure 7:**
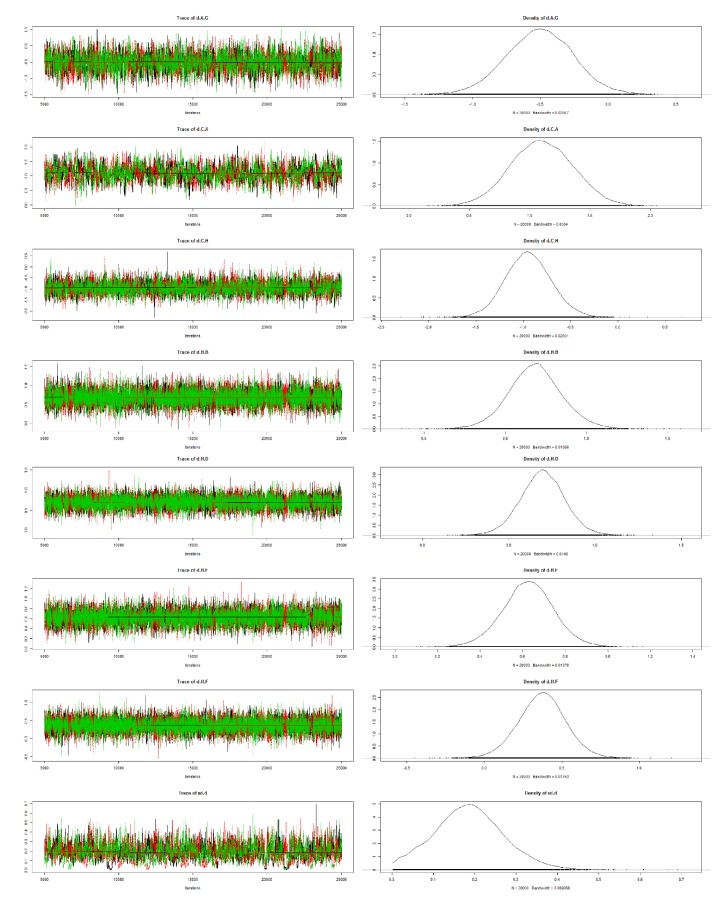
Density plot of included studies.

**Figure 8:**
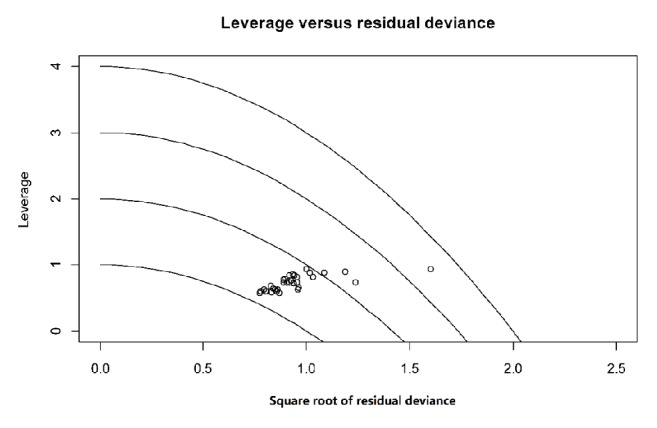
Node-splitting plot of included studies.

**Figure 9:**
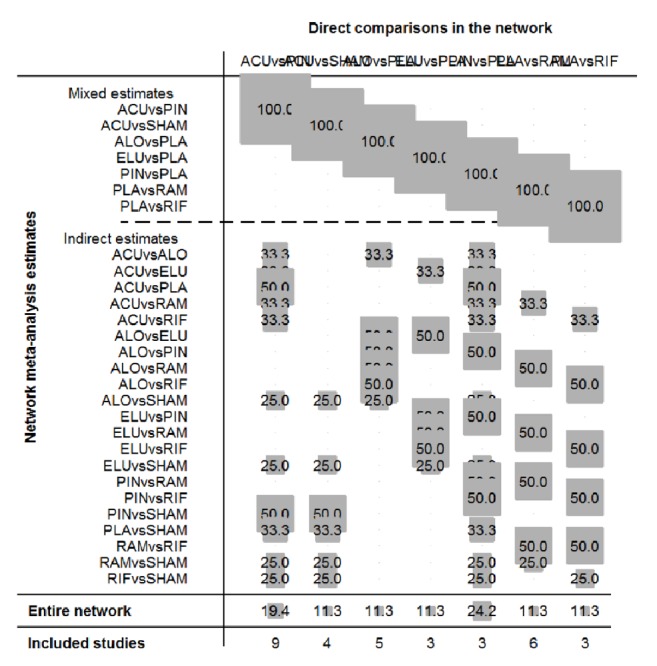
The cumulative contribution plot of IBS-D treatment of acupuncture compared with other drugs. ACU: acupuncture; ELU: eluxadoline; PIN: pinaverium bromide; ALO: alosetron; RAM: ramosetron; RIF: rifaximin; SHAM: sham acupuncture; PLA: placebo.

By performing 20,000 convergence iterations, we obtained a Brooks-Gelman-Rubin diagnostic plot, and the track density map was acceptable; based on the node-splitting model, we found all studies in the region beneath the 4th line. We also obtained a cumulative contribution map from the STATA software (Figures 6, 7, 8, and 9).

### 3.7. Heterogeneity and Sensitivity Analysis

**Figure 10:**
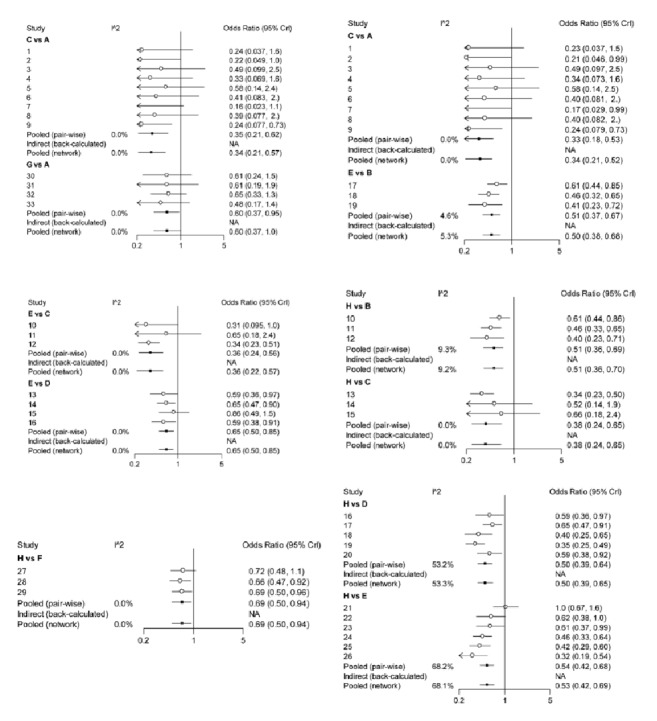
The heterogeneity analysis of included studies. A: acupuncture; B: eluxadoline; C: pinaverium bromide; D: alosetron; E: ramosetron; F: rifaximin; G: sham acupuncture; H: placebo.

Using heterogeneity analysis, we found that alosetron and ramosetron had significant heterogeneity; based on the sensitivity analysis, we corrected the OR for alosetron (OR: 1.29, 95% CI: 1.17-1.42) and the OR for placebo and ramosetron (OR: 1.33, 95% CI: 1.22-1.39), and no large directional change occurred even after corrections (Figure 10).

### 3.8. Publication Bias

**Figure 11:**
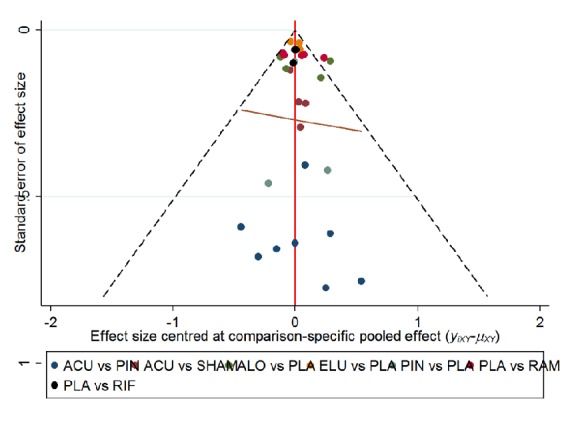
The funnel plot of all included studies. ACU: acupuncture; ELU: eluxadoline; PIN: pinaverium bromide; ALO: alosetron; RAM: ramosetron; RIF: rifaximin; SHAM: sham acupuncture; PLA: placebo.

The funnel plot shows that all included studies were compared on a pairwise basis, and all the studies were found to be essentially symmetrical, indicating a small publication bias (Figure 11).

## 4. Discussion

Through NMA, this article found that the effect of acupuncture treatment on diarrhoea-predominant irritable bowel syndrome was better than that of the assessed drugs, with close to no side effects. Previous studies have shown that the effects of acupuncture treatment on diarrhoea-predominant irritable bowel syndrome are still not yet clear, but there are several relevant studies to prove its possible role in treatment. Several studies have confirmed the co-occurrence of IBS and the excessive release of proinflammatory cytokines and insufficiencies in anti-inflammatory cytokine secretion [41]. Animal studies have shown that electroacupuncture can significantly reduce the peripheral blood flow of patients with 5-HT positive reactant content and reduce the sensitivity of afferent nerves, thereby reducing visceral hypersensitivity [42]. Studies also indicate that acupuncture can significantly reduce rat colon and dorsal root ganglia 5-HT concentrations [43]. Animal experiments have shown that acupuncture may serve as an effective treatment by regulating the abnormal state of colon mast cells [44]. Previous studies have also shown that acupuncture can reduce the number of mast EA cells in ovalbumin-sensitized mice, increasing visceral sensory thresholds and improving visceral hypersensitivity [45]. In addition, acupuncture can relieve thalamic pain in patients with advanced and central signalling pathways involving 5-HT [46]. At the same time, studies have shown that acupuncture has low side effects, an idea that has reached a certain consensus [47].

However, previous meta-analyses showed no significant benefit of acupuncture compared with sham acupuncture groups in the treatment of IBS. Only a few studies from China have demonstrated the superiority of acupuncture relative to drugs [48]. Other studies have shown that acupuncture is not or only slightly superior to sham acupuncture treatment [37]. However, our study only selected patients with IBS-D, and the effect was more significant; whether acupuncture is better for IBS-D than it is for constipation or mixed IBS remains to be further studied. A large part of this study included post-2012 studies that were inconsistent in the acupoints selected between IBS-D and other types of IBS, and this study generally included the same acupuncture points to ensure consistency in the assessment of fixed acupuncture points; to yield definitive results, sham acupuncture groups should be increased in further studies.

However, in the past, most studies conducted a direct comparison between acupuncture and pinaverium bromide. There is no direct comparison between acupuncture and other drugs such as ramosetron, alosetron, rifaximin, and eluxadoline. In the future, direct comparisons can be used to compare differences in efficacy. At the same time, this article found that the evaluation scale used in acupuncture-related research is different from other drugs (only 4 points), which will lead to a bias in the evaluation to a certain extent. In the meantime, the quantity of previous acupuncture research is relatively low, so the conclusions remain to be confirmed; these findings can be verified by increasing the sample size and using multicentre double-blind randomized controlled studies.

This study also shows sham acupuncture for the treatment of IBS-D was more effective than other drugs. Previously, there was a lack of direct comparison between sham acupuncture and oral placebo drugs, our study provides an indirect result between sham acupuncture and oral drug placebo, and there exists some curative effect for IBS-D. Actually, sham acupuncture uses the blunt needle as control, which is the same as the mechanisms of acupressure, a previous comment showed sham acupuncture may be not a good control for experiment group [49], and our study has proved this point. Now, there are many studies using sham acupuncture as control group; whether the effect of acupuncture was underestimated still needs direct comparison between sham group and oral placebo. In the future, we need to use the drug placebo control group or improve the sham acupuncture method to weaken the curative effect of sham acupuncture.

This study shows that pinaverium bromide for the treatment of IBS-D was more effective and had fewer side effects than other drugs. Previously, there was a lack of NMA comparing pinaverium bromide and other drugs. A meta-analysis of antispasmodics showed that the pinaverium bromide-induced overall improvement in symptoms of irritable bowel syndrome was 1.55 (CI 95%: 1.33-1.83) and that improvement in abdominal pain was 1.52 (CI%: 1.28-1.80) [50], which is consistent with the results obtained in this study. However, a previous study showed that the efficiency of joint pinaverium bromide-venlafaxine sustained-release tablets on IBS-D reached 85.02%, which was higher than that seen when using only pinaverium bromide (64.29%) [51]. All of the drugs compared in this study were single drugs, and this study was unable to verify multiple drug efficacies.

This study shows that alosetron has better efficacy than ramosetron, but with many side effects. Previous studies have shown the occurrence of side effects from alosetron in the treatment of IBS-D (RR = 1.16, 95% CI: 1.08, 1.25) [52], which is consistent with the results of this study. However, most patients included in our study were female patients with severe IBS-D. Alosetron is not used in the treatment of typical IBS-D patients, but for female patients with severe IBS-D, alosetron may be a good choice.

The most frequently used acupuncture points for IBS-D were ST-25, ST-37, ST-36, SP-6, GV-20, and EX-HN3. Studies have reported that the electrical stimulation of rat hind limbs at ST-36 bits can significantly improve colonic hypersensitivity [53]. Research has shown that using electroacupuncture at the ST25 stimulation site can regulate the brain glucose metabolism rates and improve visceral hypersensitivity [54]. Studies have shown that ST25 and ST37 are able to increase the pain threshold in rats with chronic visceral hypersensitivity by reducing 5-HT concentration and increasing 5-HT4R concentration [42]. Doctors choose the patient's acupuncture points based on self-judgement, preferences, and experience. It was very difficult to find consistency in previous studies, which made it difficult to achieve a consistent comparison of results because different acupoints were used. Consistent acupoint studies conducted in the future may be helpful in research or clinical applications.

This study has several advantages and disadvantages. Limitations include the poor quality of some of our studies, the relatively small number of people included, and the fact that some of the studied populations were regional. At the same time, some studies lacked safety records and some results lacked age records, which could have an impact on the results. Meanwhile, the outcome evaluation index used in this study was an overall symptom improvement scale. The drugs used in this study were single drugs. The lack of a combination effect between drugs will have a certain difference from clinical applications.

In summary, this study found that acupuncture may be a good treatment for IBS-D with few side effects, but more research is needed in the future to prove this. Sham acupuncture may be not a good control because of its curative effect for IBS-D. Pinaverium bromide is also a treatment option, as it showed a curative effect with fewer side effects.

## Figures and Tables

**Table 1:** Characteristics of included studies. NS means no available data.

Publication Date	Author	Experiment group (n)	Control group (n)	Treatments versus Control	Age of experiment group	Age of control	Diagnosis	Diagnosis criteria	Experiment Events	Control Events	Treatment Duration	Jadad	Gender	Nation	Side Effect€	Side Effect ©	Assessment tool
2015	LI Xueqing	30	30	NS/pinaverium bromide 50mg tid	46±16	44±16	IBS-D	ROME III	28	24	8 weeks	2	Mixed	China	0	0	symptom assessment tool (China) (4 points)
2014	Zhan Daowei	29	28	(LR3, ST36,SP6,ST25, ST37,GV20,EX-HN3)*∗*/pinaverium bromide 50mg tid	42±14	37±13	IBS-D	ROME III	26	19	4 weeks	3	Mixed	China	0	0	symptom assessment tool (China) (4 points)
2014	Kong Suping	29	28	(GV20,CV12, ST25, ST36, SP9, ST39)*∗*/pinaverium bromide 50mg tid	38±11	38±11	IBS-D	ROME III	26	23	4 weeks	3	Mixed	China	0	0	symptom assessment tool (China) (4 points)
2014	Liu Shuying	30	30	(GV20,EX-HN3,CV12,ST25,ST37,ST39)*∗*/pinaverium bromide 50mg tid	41.4±11.8	41.77±8.99	IBS-D	ROME III	27	23	4 weeks	1	Mixed	China	0	0	symptom assessment tool (China) (4 points)
2013	Wu Yuanjian	30	30	(ST25, ST36, ST37, SP6, CV8)*∗*/pinaverium bromide 50mg tid	37.9±10.2	39.8±11.2	IBS-D	ROME III	26	24	4 weeks	1	Mixed	China	0	0	symptom assessment tool (China) (4 points)
2012	Pei Lixia	30	30	(ST25, ST36, ST37, SP6, LR3, GV20, EX-HN3)*∗*/pinaverium bromide 50mg tid	40.9±10.6	37.93±11.45	IBS-D	ROME III	27	24	4 weeks	3	Mixed	China	0	0	symptom assessment tool (China) (4 points)
2013	LI HAO	35	35	(ST 25, ST 36, ST37, SP6, LR3, GV20, GV29)*∗*/pinaverium bromide 50mg tid	37.9±11.5	39.1±11.8	IBS-D	ROME III	33	27	4 weeks	5	Mixed	China	0	0	symptom assessment tool (China) (4 points)
2011	Sun	30	30	(ST 25, ST 36, SP6,LR3,DU20,EX-HN 3 and ST 37)*∗*/pinaverium bromide (50mg tid)	38.81±11.8	38.59±11.45	IBS-D	ROME III	27	24	4 weeks	3	Mixed	China	0	0	symptom assessment tool (China) (4 points)
2010	Shi	32	38	(ST 25, ST 36, BL 20, BL 21, BL 23, BL 25 and ST 37)*∗*/pinaverium bromide (50mg tid)	38.51±14.65	38.68±15.72	IBS-D	ROME III	26	20	4 weeks	6	Mixed	China	0	0	Overall IBS symptom VAS score (10 points)
2017	Lembo (1)	426	427	Eluxadoline 100mg /placebo BID	44.4±13.9	45.8±14.1	IBS-D	ROME III	107	73	12 weeks	7	Mixed	United States	500/859	450/808	IBS-D global symptom score, Bristol Stool Form Scale
2017	Lembo (2)	383	382	Eluxadoline 100mg /placebo BID	45.7±13.3	47.1±13.8	IBS-D	ROME III	113	62	12 weeks	7	Mixed	United States	NS	NS	IBS-D global symptom score, Bristol Stool Form Scale
2013	DOVE	163	159	Eluxadoline 100mg /placebo BID	43.6±10.9	44.6±12.5	IBS-D	ROME III	46	22	12 weeks	7	Mixed	United States	73/165	78/159	IBS Global Symptom score, IBS-SSS
2015	Liang Zheng	218	209	Pinaverium 50mg tid/placebo	36.9±11.8	36.6±12.6	IBS	ROME III	131	71	4 weeks	7	Mixed	China	40/218	32/209	Bowel Symptom Scale (10 points), Bristol stool form scale
1977	Levy	30	30	Pinaverium 50mg tid/placebo	NS	NS	IBS	Clinical	24	17	2 weeks	3	Mixed	French	NS	NS	NS
1981	Delmont	25	25	Pinaverium 50mg tid/placebo	NS	NS	IBS	Clinical	19	17	4 weeks	4	Mixed	French	NS	NS	NS
2005	Lin Chang	131	128	Alosetron 1mg/Vitamin C BID	44±12	43±12	IBS-D	ROME I	69	51	12 weeks	7	Mixed	United States	86/130	65/128	Average abdominal pain and stool consistency score (5 points)
2004	William D.Chey	279	290	Alosetron 1mg/Vitamin C BID	46.2±13.5	46.9±12.9	IBS-D	Clinical	144	119	48 weeks	7	Women	United States	297/348	261/362	Average abdominal pain and stool consistency score (5 points)
2004	Lembo (1)	147	135	Alosetron 2mg/Vitamin C BID	48.9±15.5	49.4±13.8	IBS-D	Rome II	100	62	12 weeks	6	Female	United States	145/246	127/246	IBS-D global symptom score, Average stool consistency scores (5 points)
2004	Lembo (2)	457	219	Alosetron 2mg/Vitamin C BID	48.8±14.0	48.6±13.6	IBS-D	Rome II	320	99	12 weeks	6	Female	United States	NS	NS	IBS-D global symptom score, Average stool consistency scores (5 points)
2007	Krause	177	176	Alosetron 1mg/Vitamin C BID	43	43	IBS-D	ROME II	76	54	12 weeks	7	Women	United States	102/176	94/176	IBS-D global symptom score, Average stool consistency scores (5 points)
2011	Lee KJ	175	168	Ramosetron 5ug Qd/Placebo	43.4±12.1	45±13.1	IBS-D	Rome III	65	64	4 weeks	3	Male	Korea	69/147	77/149	IBS symptoms (5 points), Bristol Stool Form Scale
2008	Matsueda (1)	297	104	Ramosetron 1ug Qd, 5ug Qd, 10ug Qd/Placebo	40.3±11.8	38.4±9.56	IBS-D	Rome II	110	28	12 weeks	6	Mixed	Japan	177/309	61/108	IBS symptoms (5 points), Bristol Stool Form Scale
2015	Fukudo S AB	307	102	Ramosetron 1.25ug Qd, 2.5ug Qd, 5ug Qd/placebo	40.9±10.6	40.2±10.1	IBS-D	Rome III	121	29	12 weeks	3	Female	Japan	NS	NS	IBS symptoms (5 points), Bristol Stool Form Scale
2016	Fukudo S	292	284	Ramosetron 2.5ug Qd/Placebo	41.4±11.8	41.5±12.0	IBS-D	Rome III	148	91	12 weeks	7	Female	Japan	154/292	118/284	IBS symptoms (5 points), Bristol Stool Form Scale
2008	Matsueda (2)	263	265	Ramosetron 5ug Qd/Placebo	40.7±11.21	41.8±11.70	IBS-D	Rome II	124	72	12 weeks	5	Mixed	Japan	163/270	141/269	IBS symptoms (5 points), Bristol Stool Form Scale
2014	Fukudo S	147	149	Ramosetron 5ug Qd/Placebo	40.9±10.6	40.2±10.1	IBS-D	Rome III	58	26	12 weeks	7	Male	Japan	13/175	6/168	IBS symptoms (5 points), Bristol Stool Form Scale
2008	Lembo	191	197	Rifaximin 550mg bid/Placebo	NS	NS	IBS-D	Rome II	100	87	2 weeks	4	Mixed	Multicenter	NS	NS	IBS-D global symptom score, IBS-associated bloating
2011	Primentel (1)	309	314	Rifaximin 550mg tid/Placebo	46.2±15.0	45.5±14.6	Non-C	Rome II	126	98	2 weeks	7	Mixed	Multicenter	264/624	296/634	IBS symptoms (5 points), Bristol Stool Form Scale
2011	Primentel (2)	315	320	Rifaximin 550mg tid/Placebo	45.9±13.9	46.3±14.6	Non-C	Rome II	128	103	2 weeks	7	Mixed	Multicenter	NS	NS	IBS symptoms (5 points), Bristol Stool Form Scale
2017	Lowe	43	36	Acupuncture/sham	42±15	43±15	IBS	Rome I	23	15	4 weeks	7	Mixed	Canada	0	0	IBS Symptoms (5 points), SF-36, IBS-36
2000	Catherine Lowe	28	22	Acupuncture/sham	NS	NS	IBS	NS	16	10	4 weeks	3	Mixed	Canada	NS	NS	NS
2009	Anthony J	78	77	Acupuncture/sham	37.5±14.6	38.9±14.1	IBS	Rome II	32	24	3 weeks	6	Female	United States	0	0	IBS-Symptom severity scale, IBS-AR, QOL
2005	Forbes	27	32	Acupuncture/sham	43	44.4	IBS	Rome+Manning	13	10	12 weeks	7	Mixed	UK	0	0	global symptom score, Bristol stool scale

**Table 2:** The cumulative probability rankings of treatment effect of acupuncture on IBS-D.

Methods/Rankings	1	2	3	4	5	6	7	8
Acupuncture	9.774500e-01	0.0221666667	0.0003166667	6.666667e-05	0.000000000	0.0000000000	0.000000000	0.000000e+00
Eluxadoline	7.000000e-04	0.0183166667	0.1130666667	3.042167e-01	0.260266667	0.2448666667	0.058383333	1.833333e-04
Pinaverium Bromide	0.000000e+00	0.0371500000	0.6937166667	1.282833e-01	0.070783333	0.0527000000	0.016733333	6.333333e-04
Alosetron	4.333333e-04	0.0141500000	0.1021333333	3.490500e-01	0.304650000	0.1983666667	0.031200000	1.666667e-05
Ramosetron	1.333333e-04	0.0056333333	0.0452000000	1.890667e-01	0.312650000	0.3764000000	0.070916667	0.000000e+00
Rifaximin	1.666667e-05	0.0005333333	0.0039000000	1.546667e-02	0.042683333	0.1198666667	0.806500000	1.103333e-02
Sham Acupuncture	2.126667e-02	0.9020500000	0.0416666667	1.385000e-02	0.008966667	0.0076833333	0.003983333	5.333333e-04
Placebo	0.000000e+00	0.0000000000	0.0000000000	0.000000e+00	0.000000000	0.0001166667	0.012283333	9.876000e-01

**Table 3:** The cumulative probability rankings of side effect of drugs on IBS-D.

Drugs/Possibility	1	2	3	4	5	6
Eluxadoline	0.0089250	0.0265875	0.082650	0.1953500	0.2967500	0.3897375
Pinaverium	0.1554125	0.1458000	0.194750	0.1840000	0.1073250	0.2127125
Alosetron	0.3657750	0.4054750	0.171675	0.0457000	0.0092500	0.0021250
Ramosetron	0.0335625	0.1281875	0.381825	0.3390875	0.0883750	0.0289625
Rifaximin	0.4363125	0.2926750	0.149800	0.0673875	0.0296375	0.0241875
Placebo	0.0000125	0.0012750	0.019300	0.1684750	0.4686625	0.3422750

**Table 4:** Most commonly used acupoints in our included articles.

Acupoint Number	Frequency	Positions
ST-25	10	Abdomen
ST-37	9	Leg
ST-36	8	Leg
SP-6	5	Leg
GV20	5	Head
EX-HN3	4	Forehead

## Supplementary Materials

Supplementary Materials Supplementary 1. Detailed searching strategy.
