# Supplementary material for: Acupuncture for Diarrhoea-Predominant Irritable Bowel Syndrome: A Network Meta-Analysis
Source: Evid Based Complement Alternat Med. 2018 May 27;2018:2890465. doi: 10.1155/2018/2890465 (PMC5994265; doi:10.1155/2018/2890465)
Supplement: Supplementary Materials — Supplementary 1. Detailed searching strategy. [file 2890465.f1.docx]

| Search ((("Irritable Bowel Syndrome"[Mesh]) OR (("Irritable Bowel Syndromes Syndrome"[Title/Abstract] OR irritable Bowel Syndromes Title/Abstract] OR irritable Bowel Colon Title/Abstract] OR irritable Irritable Colon Colitis Title/Abstract] OR mucous Colitide Title/Abstract] OR mucous Mmucous Colitides  Title/Abstract] OR "Mucous Colitis"[Title/Abstract])))) AND (("Acupuncture"[Mesh]) OR ((title/Abstract] OR Title/Abstract] OR Acupuncture H Title/Abstract] OR, Acupuncture Title/Abstract] OR acupuncture Title/Abstract] OR , Acupuncture  Title/Abstract]))) |
| --- |
| Search ("Acupuncture"[Mesh]) OR (Title/Abstract]] OR Title/Abstract] OR Acupuncture Title/Abstract] OR, Acupuncture Title/Abstract] OR acupuncture Title/Abstract] OR, Acupuncture Title/Abstract])) |
| Search (Title/Abstract] OR 18553 Title/Abstract] OR 1 Title/Abstract] OR Acupuncture Title/Abstract] OR, Acupuncture Title/Abstract] OR acupuncture Title/Abstract] OR, Acupuncture Title/Abstract]) |
| Search "Acupuncture"[Mesh] |
| Search ("Irritable Bowel Syndrome"[Mesh]) OR (("Irritable Bowel Syndromes Syndrome"[Title/Abstract] OR Irritable Bowel Syndromes Title/Abstract] OR irritable Bowel Colon Title/Abstract] OR irritable Irritable Colon Colitis Title/Abstract]  OR mucous Colitides Title/Abstract] OR mucous Mucous Colitides Title/Abstract] OR "Mmucous Colitis"[Title/Abstract])) |
| Search ("Irritable Bowel Syndrome"[Mesh]) AND (("Irritable Bowel Syndromes Syndrome"[Title/Abstract] OR irritable Bowel Syndromes Title/Abstract] OR irritable Bowel Colon Title/Abstract] OR irritable Irritable Colon Colitis Title/Abstract] OR mucous Colitides Title/Abstract] OR mucous Mmucous  Colitides Title/Abstract] OR "Mucous Colitis"[Title/Abstract])) |
| Search ("Irritable Bowel Syndromes Syndrome"[Title/Abstract] OR irritable Bowel Syndromes Title/Abstract] OR irritable Bowel Colon Title/Abstract] OR irritable Irritable Colon Colitis Title/Abstract] OR mucous Colitides Title/Abstract] OR mucous Mmucous Colitides Title/Abstract] OR "Mmucous Colitis"[Title/Abstract]) |
| Search ((("Irritable Bowel Syndrome"[Mesh]) OR (("Irritable Bowel Syndromes Syndrome"[Title/Abstract] OR irritable Bowel Syndromes Title/Abstract] OR irritable Bowel Colon Title/Abstract] OR irritable Irritable Colon Colitis Title/Abstract] OR mucous Colitide Title/Abstract] OR mucous Mmucous Colitides  Title/Abstract] OR "Mucous Colitis"[Title/Abstract])))) AND (("Eluxadoline"[Mesh]) OR ((title/Abstract] OR Title/Abstract] OR Eluxadoline H Title/Abstract] OR , Eluxadoline Title/Abstract] OR eluxadoline Title/Abstract] OR , Eluxadoline  Title/Abstract]))) |
| Search ("Eluxadoline"[Mesh]) OR (Title/Abstract]] OR Title/Abstract] OR Eluxadoline Title/Abstract] OR, Eluxadoline Title/Abstract] OR eluxadoline Title/Abstract] OR, Eluxadoline Title/Abstract])) |
| Search (Title/Abstract] OR 18553 Title/Abstract] OR 1 Title/Abstract] OR Eluxadoline Title/Abstract] OR, Eluxadoline Title/Abstract] OR eluxadoline Title/Abstract] OR, Eluxadoline Title/Abstract]) |
| Search "Eluxadoline"[Mesh] |
| Search ("Irritable Bowel Syndrome"[Mesh]) OR (("Irritable Bowel Syndromes Syndrome"[Title/Abstract] OR Irritable Bowel Syndromes Title/Abstract] OR irritable Bowel Colon Title/Abstract] OR irritable Irritable Colon Colitis Title/Abstract]  OR mucous Colitides Title/Abstract] OR mucous Mucous Colitides Title/Abstract] OR "Mmucous Colitis"[Title/Abstract])) |
| Search ("Irritable Bowel Syndrome"[Mesh]) AND (("Irritable Bowel Syndromes Syndrome"[Title/Abstract] OR irritable Bowel Syndromes Title/Abstract] OR irritable Bowel Colon Title/Abstract] OR irritable Irritable Colon Colitis Title/Abstract] OR mucous Colitides Title/Abstract] OR mucous Mmucous  Colitides Title/Abstract] OR "Mucous Colitis"[Title/Abstract])) |
| Search ("Irritable Bowel Syndromes Syndrome"[Title/Abstract] OR irritable Bowel Syndromes Title/Abstract] OR irritable Bowel Colon Title/Abstract] OR irritable Irritable Colon Colitis Title/Abstract] OR mucous Colitides Title/Abstract] OR mucous Mmucous Colitides Title/Abstract] OR "Mmucous Colitis"[Title/Abstract]) |
| Search "Irritable Bowel Syndrome"[Mesh] |
| Search ((("Irritable Bowel Syndrome"[Mesh]) OR (("Irritable Bowel Syndromes Syndrome"[Title/Abstract] OR irritable Bowel Syndromes Title/Abstract] OR irritable Bowel Colon Title/Abstract] OR irritable Irritable Colon Colitis Title/Abstract] OR mucous Colitide Title/Abstract] OR mucous Mmucous Colitides  Title/Abstract] OR "Mucous Colitis"[Title/Abstract])))) AND (("Pinaverium bromide"[Mesh]) OR ((title/Abstract] OR Title/Abstract] OR Pinaverium bromide Title/Abstract] OR , Pinaverium bromide Title/Abstract] OR Pinaverium bromide Title/Abstract] OR , Pinaverium bromide  Title/Abstract]))) |
| Search ("Pinaverium bromide"[Mesh]) OR (Title/Abstract]] OR Title/Abstract] OR Pinaverium bromide Title/Abstract] OR , Pinaverium bromide Title/Abstract] OR Pinaverium bromide Title/Abstract] OR, Pinaverium bromide Title/Abstract])) |
| Search (Title/Abstract] OR 18553 Title/Abstract] OR 1 Title/Abstract] OR Pinaverium bromide Title/Abstract] OR, Pinaverium bromide Title/Abstract] OR Pinaverium bromide Title/Abstract] OR, Pinaverium bromide Title/Abstract]) |
| Search "Pinaverium bromide"[Mesh] |
| Search ("Irritable Bowel Syndrome"[Mesh]) OR (("Irritable Bowel Syndromes Syndrome"[Title/Abstract] OR Irritable Bowel Syndromes Title/Abstract] OR irritable Bowel Colon Title/Abstract] OR irritable Irritable Colon Colitis Title/Abstract]  OR mucous Colitides Title/Abstract] OR mucous Mucous Colitides Title/Abstract] OR "Mmucous Colitis"[Title/Abstract])) |
| Search ("Irritable Bowel Syndrome"[Mesh]) AND (("Irritable Bowel Syndromes Syndrome"[Title/Abstract] OR irritable Bowel Syndromes Title/Abstract] OR irritable Bowel Colon Title/Abstract] OR irritable Irritable Colon Colitis Title/Abstract] OR mucous Colitides Title/Abstract] OR mucous Mmucous  Colitides Title/Abstract] OR "Mucous Colitis"[Title/Abstract])) |
| Search ("Irritable Bowel Syndromes Syndrome"[Title/Abstract] OR irritable Bowel Syndromes Title/Abstract] OR irritable Bowel Colon Title/Abstract] OR irritable Irritable Colon Colitis Title/Abstract] OR mucous Colitides Title/Abstract] OR mucous Mmucous Colitides Title/Abstract] OR "Mmucous Colitis"[Title/Abstract]) |
| Search "Irritable Bowel Syndrome"[Mesh] |
| Search ((("Irritable Bowel Syndrome"[Mesh]) OR (("Irritable Bowel Syndromes Syndrome"[Title/Abstract] OR irritable Bowel Syndromes Title/Abstract] OR irritable Bowel Colon Title/Abstract] OR irritable Irritable Colon Colitis Title/Abstract] OR mucous Colitide Title/Abstract] OR mucous Mmucous Colitides  Title/Abstract] OR "Mucous Colitis"[Title/Abstract])))) AND (("Alosetron"[Mesh]) OR ((title/Abstract] OR Title/Abstract] OR Alosetron Title/Abstract] OR , Alosetron Title/Abstract] OR Alosetron Title/Abstract] OR , Alosetron  Title/Abstract]))) |
| Search ("Alosetron"[Mesh]) OR (Title/Abstract]] OR Title/Abstract] OR Alosetron Title/Abstract] OR , Alosetron Title/Abstract] OR Alosetron Title/Abstract] OR, Alosetron Title/Abstract])) |
| Search (Title/Abstract] OR 18553 Title/Abstract] OR 1 Title/Abstract] OR Alosetron Title/Abstract] OR, Alosetron Title/Abstract] OR Alosetron Title/Abstract] OR, Alosetron Title/Abstract]) |
| Search "Alosetron"[Mesh] |
| Search ("Irritable Bowel Syndrome"[Mesh]) OR (("Irritable Bowel Syndromes Syndrome"[Title/Abstract] OR Irritable Bowel Syndromes Title/Abstract] OR irritable Bowel Colon Title/Abstract] OR irritable Irritable Colon Colitis Title/Abstract]  OR mucous Colitides Title/Abstract] OR mucous Mucous Colitides Title/Abstract] OR "Mmucous Colitis"[Title/Abstract])) |
| Search ("Irritable Bowel Syndrome"[Mesh]) AND (("Irritable Bowel Syndromes Syndrome"[Title/Abstract] OR irritable Bowel Syndromes Title/Abstract] OR irritable Bowel Colon Title/Abstract] OR irritable Irritable Colon Colitis Title/Abstract] OR mucous Colitides Title/Abstract] OR mucous Mmucous  Colitides Title/Abstract] OR "Mucous Colitis"[Title/Abstract])) |
| Search ("Irritable Bowel Syndromes Syndrome"[Title/Abstract] OR irritable Bowel Syndromes Title/Abstract] OR irritable Bowel Colon Title/Abstract] OR irritable Irritable Colon Colitis Title/Abstract] OR mucous Colitides Title/Abstract] OR mucous Mmucous Colitides Title/Abstract] OR "Mmucous Colitis"[Title/Abstract]) |
| Search "Irritable Bowel Syndrome"[Mesh] |
| Search ((("Irritable Bowel Syndrome"[Mesh]) OR (("Irritable Bowel Syndromes Syndrome"[Title/Abstract] OR irritable Bowel Syndromes Title/Abstract] OR irritable Bowel Colon Title/Abstract] OR irritable Irritable Colon Colitis Title/Abstract] OR mucous Colitide Title/Abstract] OR mucous Mmucous Colitides  Title/Abstract] OR "Mucous Colitis"[Title/Abstract])))) AND (("Ramosetron"[Mesh]) OR ((title/Abstract] OR Title/Abstract] OR Ramosetron Title/Abstract] OR , Ramosetron Title/Abstract] OR Ramosetron Title/Abstract] OR , Ramosetron  Title/Abstract]))) |
| Search ("Ramosetron"[Mesh]) OR (Title/Abstract]] OR Title/Abstract] OR Ramosetron Title/Abstract] OR , Ramosetron Title/Abstract] OR Ramosetron Title/Abstract] OR, Ramosetron Title/Abstract])) |
| Search (Title/Abstract] OR 18553 Title/Abstract] OR 1 Title/Abstract] OR Ramosetron Title/Abstract] OR, Ramosetron Title/Abstract] OR Ramosetron Title/Abstract] OR, Ramosetron Title/Abstract]) |
| Search "Ramosetron"[Mesh] |
| Search ("Irritable Bowel Syndrome"[Mesh]) OR (("Irritable Bowel Syndromes Syndrome"[Title/Abstract] OR Irritable Bowel Syndromes Title/Abstract] OR irritable Bowel Colon Title/Abstract] OR irritable Irritable Colon Colitis Title/Abstract]  OR mucous Colitides Title/Abstract] OR mucous Mucous Colitides Title/Abstract] OR "Mmucous Colitis"[Title/Abstract])) |
| Search ("Irritable Bowel Syndrome"[Mesh]) AND (("Irritable Bowel Syndromes Syndrome"[Title/Abstract] OR irritable Bowel Syndromes Title/Abstract] OR irritable Bowel Colon Title/Abstract] OR irritable Irritable Colon Colitis Title/Abstract] OR mucous Colitides Title/Abstract] OR mucous Mmucous  Colitides Title/Abstract] OR "Mucous Colitis"[Title/Abstract])) |
| Search ("Irritable Bowel Syndromes Syndrome"[Title/Abstract] OR irritable Bowel Syndromes Title/Abstract] OR irritable Bowel Colon Title/Abstract] OR irritable Irritable Colon Colitis Title/Abstract] OR mucous Colitides Title/Abstract] OR mucous Mmucous Colitides Title/Abstract] OR "Mmucous Colitis"[Title/Abstract]) |
| Search "Irritable Bowel Syndrome"[Mesh] |
| Search ((("Irritable Bowel Syndrome"[Mesh]) OR (("Irritable Bowel Syndromes Syndrome"[Title/Abstract] OR irritable Bowel Syndromes Title/Abstract] OR irritable Bowel Colon Title/Abstract] OR irritable Irritable Colon Colitis Title/Abstract] OR mucous Colitide Title/Abstract] OR mucous Mmucous Colitides  Title/Abstract] OR "Mucous Colitis"[Title/Abstract])))) AND (("Rifaximin"[Mesh]) OR ((title/Abstract] OR Title/Abstract] OR Rifaximin Title/Abstract] OR , Rifaximin Title/Abstract] OR Rifaximin Title/Abstract] OR , Rifaximin  Title/Abstract]))) |
| Search ("Rifaximin"[Mesh]) OR (Title/Abstract]] OR Title/Abstract] OR Rifaximin Title/Abstract] OR , Rifaximin Title/Abstract] OR Rifaximin Title/Abstract] OR, Rifaximin Title/Abstract])) |
| Search (Title/Abstract] OR 18553 Title/Abstract] OR 1 Title/Abstract] OR Rifaximin Title/Abstract] OR, Rifaximin Title/Abstract] OR Rifaximin Title/Abstract] OR, Rifaximin Title/Abstract]) |
| Search "Rifaximin"[Mesh] |
| Search ("Irritable Bowel Syndrome"[Mesh]) OR (("Irritable Bowel Syndromes Syndrome"[Title/Abstract] OR Irritable Bowel Syndromes Title/Abstract] OR irritable Bowel Colon Title/Abstract] OR irritable Irritable Colon Colitis Title/Abstract]  OR mucous Colitides Title/Abstract] OR mucous Mucous Colitides Title/Abstract] OR "Mmucous Colitis"[Title/Abstract])) |
| Search ("Irritable Bowel Syndrome"[Mesh]) AND (("Irritable Bowel Syndromes Syndrome"[Title/Abstract] OR irritable Bowel Syndromes Title/Abstract] OR irritable Bowel Colon Title/Abstract] OR irritable Irritable Colon Colitis Title/Abstract] OR mucous Colitides Title/Abstract] OR mucous Mmucous  Colitides Title/Abstract] OR "Mucous Colitis"[Title/Abstract])) |
| Search ("Irritable Bowel Syndromes Syndrome"[Title/Abstract] OR irritable Bowel Syndromes Title/Abstract] OR irritable Bowel Colon Title/Abstract] OR irritable Irritable Colon Colitis Title/Abstract] OR mucous Colitides Title/Abstract] OR mucous Mmucous Colitides Title/Abstract] OR "Mmucous Colitis"[Title/Abstract]) |
| Search "Irritable Bowel Syndrome"[Mesh] |
|  |
